# Breeding System of *Jacquemontia curtisii*, an Endemic Morning-Glory of Pine Rocklands

**DOI:** 10.3390/biology15181595

**Published:** 2026-09-10

**Authors:** Suzanne Koptur

**Affiliations:** Department of Biological Sciences, International Center for Tropical Botany, Institute of the Environment, Florida International University, Miami, FL 33199, USA; kopturs@fiu.edu; Tel.: +1-305-984-0539

**Keywords:** Convolvulaceae, hand-pollination, imperiled plant, nectar, pine rocklands, self-incompatibility, state-threatened taxon

## Abstract

The pine rocklands of southern Florida are a globally imperiled ecosystem, and as a result many species endemic to this habitat are endangered or threatened. To help conserve the endemic plants of the imperiled pine rockland habitat in southern Florida, it is important to understand factors that influence their successful reproduction. Field-collected individuals of *Jacquemontia curtisii* Peter ex Hallier f. (Convolvulaceae), a threatened species of perennial herb, were grown in a greenhouse to control conditions for floral measurements and hand-pollinations. While flowering lasts for months, individual flowers last one day, open from dawn through mid-morning, and produce nectar as reward for pollinators. Each flower contains 0.25–1 μL of nectar, with 22–50% sugar on a wt/wt basis; larger nectar volumes usually corresponded to lower sugar concentrations. Plants with larger flowers have smaller nectar volumes than those with smaller flowers, but higher nectar sugar concentrations. Flowers were subjected to one of four pollination treatments: not manipulated (autonomous selfing), anthers removed (emasculation), selfed, or crossed. Five or ten flowers on each plant received each treatment, except for crosses, where ten to twenty flowers were used, crossed with pollen from different individuals. The results show the species to be highly self-incompatible, but some individuals (one-fifth of the plants studied) are able to set fruit also with their own pollen. Fruit set observed in the field can be interpreted as evidence of pollinator activity with successful transfer of pollen from mostly other compatible individuals. There is therefore a danger of species decline if small habitat fragments contain only individuals that are closely related.

## Simple Summary

When studying plants that are endangered or threatened with extinction, it is essential to understand their reproductive biology. We undertook this study to learn how pollen source affects pollination success in a morning-glory found only in pine rocklands. Flowers of these plants are much more likely to produce fruit if they are cross-pollinated, indicating that without a sufficient number of other individuals that are not closely related, the populations may decline. Without successful pollination and production of fruits and seeds, plant populations cannot persist. Pine rocklands in south Florida have been greatly reduced by human activities (clearing and building on the rockland ground), and plants found only in this habitat may suffer if they are unable to mate with compatible individuals.

## 1. Introduction

The pine rocklands of southern Florida occur on the highest, driest land in a very low elevation landscape growing on a foundation of limestone relatively recently exposed from receding seas [1]. Consequently, much of this habitat has been destroyed by human development as people sought to build homes, businesses, and farms on this higher ground [2,3]. Pine rocklands are a fire successional habitat, with a diverse flora of herbaceous plants, many of which are endemic, under a canopy of Dade County slash pine (*Pinus elliottii* var. *densa*) maintained by fire every 5–15 years that prevents overgrowth by subtropical hardwood species [4,5]. Outside of Everglades National Park, less than 2% of the original extent along the Miami Rock Ridge remains, as fragments of different sizes, from 1 ha to more than 2500 ha [3,6]. As part of a larger study to determine the effects of habitat fragmentation on pollination and reproduction of pine rockland plants, we studied the breeding systems, floral biology, and pollination ecology of an array of flowering plants in different families with diverse flower shapes and sizes [2]. Here we elucidate the breeding system of a distinctive, emblematic pine rockland species, *Jacquemontia curtisii* Peter ex Hallier f. (Convolvulaceae), the pineland cluster vine.

Due to the extensive loss of habitat, pine rocklands are considered globally critically imperiled by the Florida Natural Areas Inventory (FNAI) [7], and many pine rockland endemic animals, including Bartram’s hairstreak and Florida leafwing butterflies [8], the Bonneted Bat and the Miami Tiger Beetle [9,10], and the Rim-Rock Crowned and Key Ring-Necked snakes [11], are taxa of conservation concern. Numerous pine rockland plant species of concern are endemic to pine rocklands and occur in habitat fragments [12], including endangered *Galactia* spp. and *Euphorbia* (*Chamaesyce*) spp. [13], *Amorpha crenulata* [14], *Polygala smallii*, *Ipomoea microdactyla* [15], *Linum arenicola* [16], and *Chamaecrista lineata* var. *keyensis* [17].

To fully understand whether flower visitors are effective pollinators, it is important not only to know if they transfer pollen to stigmas but also to know if individual plants can set fruit with their own pollen (are self-compatible) or if they need to receive pollen from another individual (are self-incompatible). Understanding a species’ breeding system is essential when studying potential negative effects of habitat fragmentation [18,19,20,21,22,23,24] or other disturbances such as fire [25,26,27,28], pesticides [29,30,31], and hurricanes [25,32,33,34].

In this study we examined the breeding system of an iconic plant of south Florida pine rocklands, *Jacquemontia curtisii*, the pineland clustervine, testing self-compatibility and self-incompatibility. While its pollination has been studied in the field, its breeding system was unknown. We therefore asked: are *Jacquemontia curtisii* plants self-compatible, or do they need pollen from other individuals to produce fruits and seeds?

## 2. Materials and Methods

### 2.1. Study System

**Figure 1:**
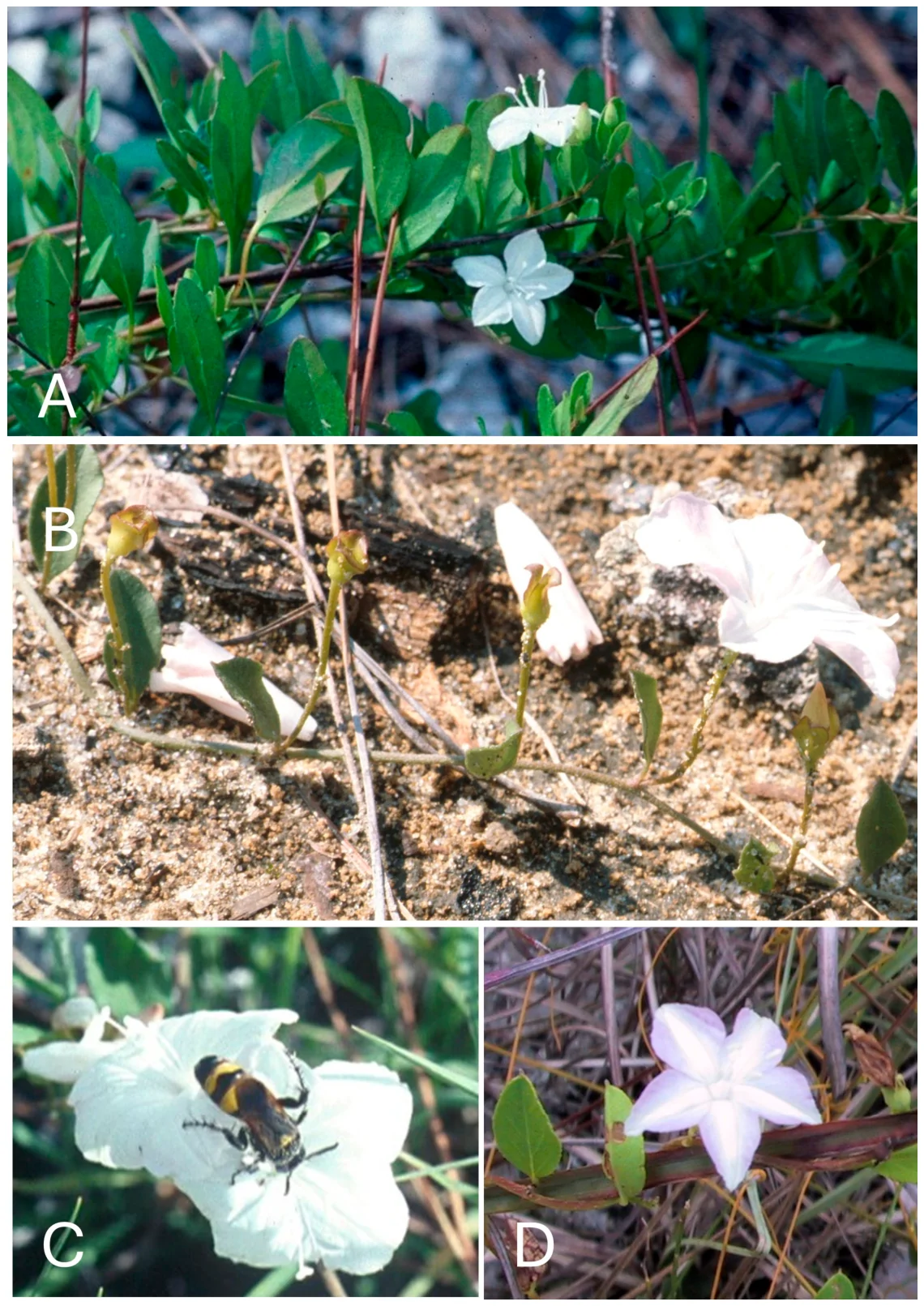
(**A**) *Jacquemontia curtisii* habit growing along pine rockland surface; (**B**) growing on sandy soil, immature fruits are visible to left, fallen corollas from the previous day’s flowers, an open flower, and a bud for the next day’s flower to the right; (**C**) flower visitor contacting stamens and stigma while taking nectar; (**D**) pink-edged color form of flower.

*Jacquemontia curtisii*, the pineland cluster vine, has bright white flowers spilling from its stems that climb over other plants in the understory or along the ground (Figure 1A,B); some individuals have petals tipped with pink or green, as shown in Figure 1D. As do many other pine rockland understory plants, *J. curtisii* depends on openings provided by fire [3,35] and other disturbances to thrive and reproduce.

While not federally listed as an endangered species, *Jacquemontia curtisii* is considered imperiled by Natureserve, the Institute for Regional Conservation (IRC), and FNAI and threatened by the State of Florida, as it is frequent only in pine rockland fragments on the Miami Rock Ridge. Its generalist, open flowers are visited by a wide array of insects (Figure 1C), including bees, wasps, flies, and butterflies [36]. Visitors to flowers collect nectar or pollen, and field studies have shown that stigmas of flowers receive a substantial amount of *J. curtisii* pollen as well as pollen of other sympatric plant species [36].

### 2.2. Experimental Procedure

For this investigation, we used plants collected from the field. At a privately owned site facing imminent development, we were able to rescue the population of *Jacquemontia curtisii* in April 1999. We excavated more than thirty individuals from a large meadow that was then cleared and paved for a parking lot.

We brought the plants to the greenhouse at Florida International University in Miami, Florida, USA, where we cut back the shoots to allow the plants to recover from their transplanting. Within two months the plants had grown new shoots and began to flower, and we undertook our measurements and experiments.

Flowers opened each morning by 7 am and lasted for only one day. By the late afternoon, or the following morning, the corolla had fallen and could be found below the plant (Figure 1B). It was also possible to see which buds would open the next day.

For each of 30 plants, we collected and measured 30 open flowers, examining them under a dissecting microscope and recording corolla length, corolla lobe length (beyond the point of sympetaly), stamen length (of free filament from base to top of anthers), and style length. We subtracted stamen length from style length to obtain values for style-stamen separation. Means of flower measurements for all plants were obtained.

On a subset of eleven individuals, we measured nectar volume and concentration in multiple (20–30) flowers on each plant, using 1 μL micropipettes to extract and quantify the nectar contained in each flower and a hand-held refractometer (non-digital, adjusted for small quantities; Bellingham and Stanley brand, Tunbridge Wells, UK, now a brand under Xylem Analytics, Weilheim, Germany) to measure sugar concentration on a weight/weight basis for each nectar sample. We averaged the values among the flowers to obtain mean values of these parameters for each plant.

We conducted controlled hand-pollinations in this controlled environment, replicating four treatments on ten flowers each on 340 individual plants. We performed five or more additional cross-pollinations per plant, using different pollen parents; therefore, twenty or more flowers per plant were used. We did not bag flowers, as the greenhouse was assumed to be pollinator-free. Each flower was marked individually using a paper jewelry tag recording the treatment and (where relevant) pollen parent. Although plants with pink- or green-tinged flowers do occur (Figure 1D), all the plants in this experiment had white corollas. All pollination treatments were performed in the early mornings between 7 and 10 am on flowers newly open that morning.

The treatments were:

(1) autonomous self-pollination—flowers were tagged and not otherwise manipulated;
(2) emasculation—anthers were removed from stamens prior to dehiscence to prevent pollen from reaching stigma to test for seed formation without fertilization;
(3) self-pollination—pollen from a flower was moved to its own stigma using fine forceps to take an anther and apply its pollen to the stigmatic surface; and
(4) cross-pollination—pollen from another individual plant was deposited on the stigma using forceps to apply pollen by rubbing its anther on the stigma of the recipient flower.

In all cases of self- and cross-pollination we examined the stigmas with a hand lens to ensure that pollen was transferred. We cleaned our forceps with alcohol between each pollination to prevent contamination. We performed multiple cross-pollinations for each plant, using different individuals as pollen parents. Flowers in all treatments were tagged individually and followed to see which had set fruit. Fruits matured from green to brown in three to four weeks. Fruits were collected individually to count and weigh the seeds produced.

### 2.3. Statistical Procedure

We examined the nectar data to see if a relationship existed between volume and concentration among all the plants using Pearson’s correlation analysis. We examined variation in flower measurements among individuals using GLM multivariate analysis of variance. As we had these measurements for ten of the individuals for which we measured nectar, we used Pearson’s correlation to examine the relationship between corolla size and nectar volume and nectar concentration.

We compared fruit set among treatments on all plants using 2 × c contingency table analyses. We used a 2 × 2 contingency table to compare fruit set of self-pollinated vs. cross-pollinated flowers on each individual plant, as well as on overall totals. We weighed individual seeds produced by self- vs. cross-pollinations to see if there was a difference between the treatments. As there were very few fruits from self-pollinations and therefore a great disparity in sample sizes, we used the non-parametric Kruskal–Wallis test. Statistical analyses were performed using IBM SPSS version 29.0.1.1.

## 3. Results

### 3.1. Flower Measurements

Flower size varied by individual plant (Table 1), with a potential difference in corolla length between plants of nearly 1 cm. Average corolla lobe length was less than half the corolla length, and styles were longer than stamens in all flowers. Stigmas were therefore always slightly higher than the anthers, with the separation between them being on average 0.2 cm (range: 0.09 cm to 0.38 cm). Multivariate analysis of variance (with type 2 sum of squares and df = 31) showed all measurements differing significantly among plants: corolla length (F = 16.64, *p* < 0.001); petal length (F = 16.43, *p* < 0.001); stamen length (F = 10.58, *p* < 0.001); and style length (F = 11.94, *p* < 0.001).

### 3.2. Nectar Production Variation Among Individuals

**Figure 2:**
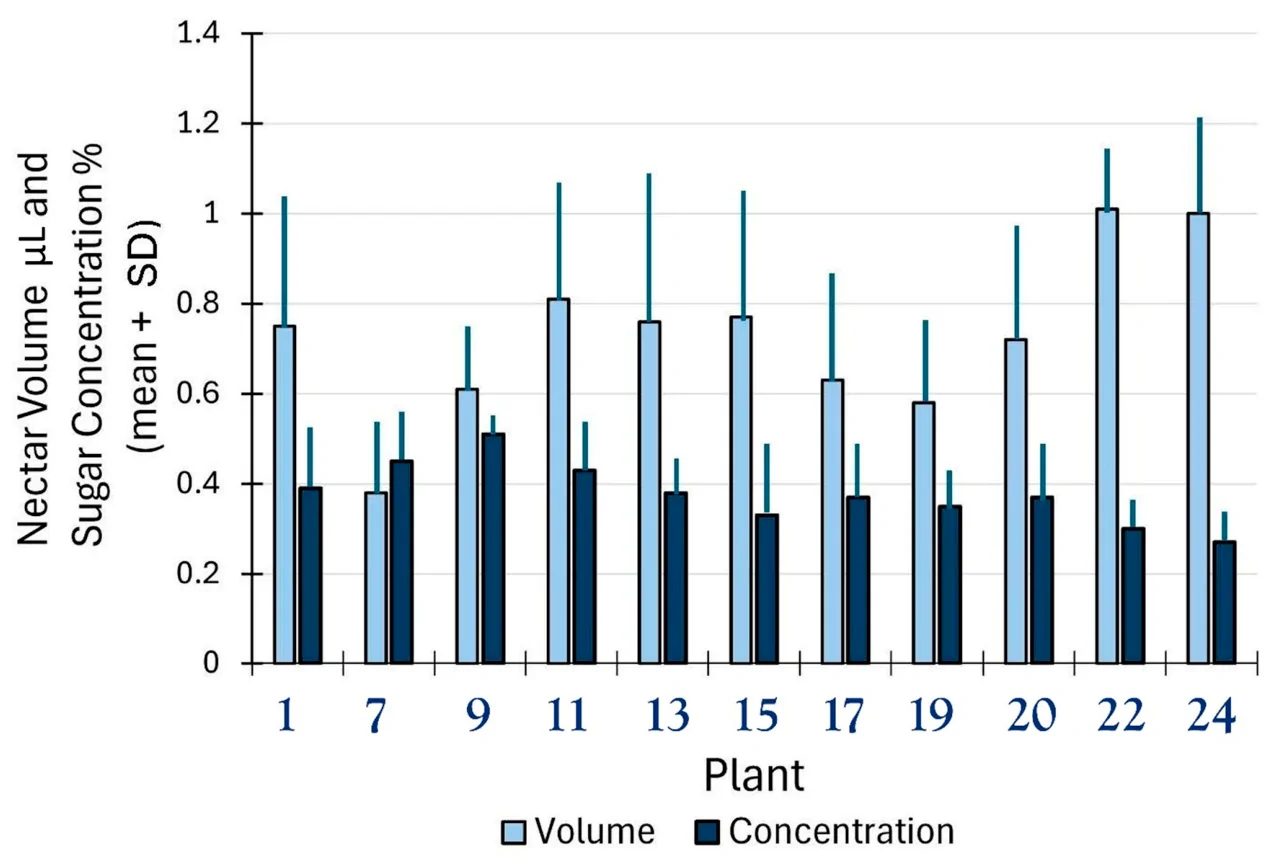
Nectar volume and sugar concentrations (mean + SE) of individual flowers on eleven greenhouse grown individuals (Plant ID number shown for each) of *Jacquemontia curtisii*. As flowers open in the morning and last a single day, these are for the total nectar content produced per flower. Nectar volumes per plant were inversely correlated with sugar concentration (r = −0.40, *p* < 0.01).

Nectar volume per flower was on average 0.76 ± 0.28 μL (range: 0.6 μL to 1.05 μL per plant), and sugar concentrations were 35% ± 11% (range: 25–56% per plant). Regression showed a slight inverse correlation (r = −0.40, *p* < 0.01) between nectar volume and concentration (Figure 2), with plants whose flowers had greater nectar volumes often having lower sugar concentrations than those with smaller nectar volumes (Figure 2). There was a negative relationship between mean flower size (corolla length) and mean nectar volume per flower per plant (r =−0.47) and a positive relationship between size and nectar concentration (r = 0.61).

### 3.3. Hand-Pollination Experiments

Overall, *J. curtisii* plants set much more fruit with cross-pollination than self-pollination (82% vs. 6%; Table 2). Only seven of the thirty-four plants showed some self-compatibility, setting fruit with both self- and cross-pollen; 27 plants were entirely self-incompatible (Table S1). Thirty-two of 34 plants showed significantly greater fruit set with cross-pollination than self-pollination (Table S1). A very small percentage of flowers set fruit with no manipulation (3/364 = < 1%), an indication perhaps of a very small amount of autonomous selfing; there was also a small amount of fruit set in flowers with anthers removed (2/360 = < 0.01%). Both of these treatment results may have been experimental error (see Discussion). Contingency table analysis of all treatments shows a highly significant difference between crosses and other treatments (Pearson’s X^2^_(3)_ = 359.1, *p* < 0.001). Comparing only crosses and selfs, this difference is also highly significant (Pearson X^2^_(1)_ = 587.47, *p* < 0.0001).

### 3.4. Seed Mass Comparison

We compared the weights of seeds produced by self-pollination (*n* = 14) with those resulting from cross-pollination (*n* = 557). The difference in mean seed mass was not significant over all treatments (Kruskal–Wallis X^2^_3_ = 9.35, *p* = 0.25), nor was it when comparing seeds from cross- vs. self-pollinations (Kruskal–Wallis X^2^_1_ = 1.25, *p* = 0.265; Table 3).

## 4. Discussion

Diurnally opening white flowers are strikingly visible against the green vegetation of the pine rockland, and earlier work has shown that the flowers of *Jacquemontia curtisii* are visited by a wide array of floral visitors that are rewarded with both pollen and nectar. The nectar is easily accessed by a variety of visitors [36], with an average sugar concentration of 35% and average volume of less than one μL (0.76 μL) per flower. Average nectar concentrations varied among individual plants (from 30 to 50% sugar on a wt/wt basis), and there was a negative correlation between average sugar concentration and average nectar volume per plant. Larger flowers had lower nectar volumes than smaller flowers, but their nectar was more concentrated.

The concentration of nectar in the flowers is typical for bee-pollinated species [37,38], and the amount of nectar is comparable to other mid-sized flowers: less than the amount provided by *Ruellia succulenta* (Acanthaceae) [39] (ca. 1 μL per flower and mean concentration 19.6%), *Angadenia berteroi* (Apocynaceae) (ca. 1.5 μL per flower and mean concentration 44%) [40] that are visited by both butterflies and bees, and *Centrosema virginiana* (Fabaceae) [41] (up to 1.8 μL per flower and mean concentration 40%, visited by bees exclusively).

Some plant species of the pine rockland set fruit with no pollinator visits, with most individuals having the ability to self-pollinate automatically [16,39]. Some need pollinator visits but are entirely self-compatible [42]. Like numerous other pine rockland plant species [14,40,43,44], *Jacquemontia curtisii* is largely self-incompatible, setting substantially more fruit with cross- versus self-fertilization. As a small percentage of individuals in the studied population set some fruit with self-pollination, a small percentage of fruit set observed in the field is likely the result of self-pollination; however, as *J. curtisii* did not display much autonomous self-pollination (2%), most fruit set observed in the field may be interpreted as evidence of pollinator visits that transfer pollen between flowers on the same or different plants.

*Jacquemontia* species that have been studied in other habitats are self-compatible to different degrees: *J. nodiflora* had a high rate of automatic selfing that was equal to hand-self-pollinations (90% or more) and even greater than cross-pollinations [45]. *J. bracteosa* also set a large amount of fruit with hand-self-pollination (30–80%), slightly lower than 93% fruit set with hand-cross-pollination [46]. In the Brazilian caatinga, *J. multiflora* was similarly facultatively outcrossing, with 30% fruit set from selfing and 60% from cross-pollination [47]. The other south Florida species that was studied with controlled hand-pollinations, *J. reclinata* [48], showed no autonomous selfing but greater self-compatibility (20% fruit set with hand-self-pollinations) than did *J. curtisii* (only 6%). Our observation of 2% fruit set with no floral manipulation suggests that autonomous selfing may exist at a very low level in *J. curtisii*, but the fact that one emasculated flower also set fruit set reveals this may have been the result of experimental error and/or our assumption that the greenhouse was pollinator-free.

*Jacquemontia curtisii* appears to be more self-incompatible than all other species of *Jacquemontia* so far studied. Pollen/ovule ratios measured of the south Florida *Jacquemontia* species predicted this might be the case [36], as the P/O ratio of *J. curtisii* was more than twice that of *J. reclinata* (approximately 4000:1 vs. 1600:1). In general, larger P/O ratios are seen in flowering plants that display more xenogamy vs. autogamy [49,50,51], though pollen transfer efficiency also plays a role [52]. Furthermore, breeding systems may vary among populations of the same species [53,54,55], so we may have an incomplete picture at this point, considering we studied plants from only one population. Many plants display mixed-mating strategies [56], reproducing by both self- and cross-pollination; nearly half of all animal-pollinated species employ mixed-mating. Despite inbreeding depression that may result from selfing, a plant that is able to reproduce with self-pollination will still be able to produce some seed when there are few or no potential mates around.

Self-compatibility varies within a population as well as between populations [57], and self-pollinating species often have smaller and less rewarding flowers than those of their outcrossing counterparts [58,59]. In comparison to other pine rockland plants in the same family, Convolvulaceae, *Jacquemontia curtisii* has flowers that are intermediate in size: less than half the size of *Ipomoea microdactyla* flowers, and more than twice as large as those of *Evolvulus sericeus*. Its flowers are twice the size of those of the highly self-compatible *J. nodiflora* [45], slightly larger than flowers of *J. multiflora* [47], similar in size to those of *J. reclinata* [48] and *J. montana* [60], and smaller than those of *J. bracteosa* [46].

Previous work has shown that almost every flower of *Jacquemontia curtisii* in the field receives *J. curtisii* pollen on its stigma [36], as well as pollen of other co-blooming sympatric plants, as expected for a generalist pollinated species. Our findings here indicate that if that pollen is not from another *J. curtisii* individual, there may be little chance of fertilization or fruit set. This finding is valuable for interpreting fruit set in the field, especially in comparing remnant pine rockland sites of different sizes and degrees of isolation.

## 5. Conclusions

Using hand-pollination experiments on field-collected individuals from one population, we have demonstrated that *Jacquemontia curtisii* is largely self-incompatible. Some individuals, however, can set fruit with self-pollination, though at a much lower rate than with cross-pollination. This strategy may permit a small population to persist through times when pollinators are limited or when there are few other individual plants to serve as potential mates. In conserving these threatened plants of the imperiled pine rockland habitat, we conclude that it is important to maintain areas large enough to hold an adequate number of individuals to allow for pollinator attraction, pollination, and seed production necessary for the persistence of their populations.

## Figures and Tables

**Table 1:** Flower dimensions of *Jacquemontia curtisii*. Presented are means of individual plant averages (20 flowers per 30 plants, overall *n* = 635 flowers).

Parameter	Mean ± S.D. (cm)	Range (cm)
Corolla length	2.75 ± 0.28	2.34–3.18
Petal length	1.35 ± 0.15	1.18–1.55
Stamen length	0.89 ± 0.10	0.82–0.99
Style length	1.12 ± 0.12	0.92–1.26
Stigma–Anther separation	0.22 ± 0.11	0.09–0.38

**Table 2:** Summary of hand-pollination fruit production results. The number of flowers in each treatment that produced fruit (and did not) are shown, as well as the total number of flowers receiving each treatment. % fruit set was determined by dividing the number of fruits by the number of flowers pollinated. More crosses were performed than the other treatments, as we used pollen from multiple single-parent plants for each cross. ** Signifies significant difference at the <0.0001 level, between self- and cross-pollination fruit set.

Total of All Plants (N = 34)							
Plant	Cross	Fruit	No Fruit	#Flrs	% Fruit Set	X^2^_1_	*p*
	Self	22	346	368	6		
	Cross	618	133	751	82	587.47	0.0000 **
	Total	640	479	1119	-		

Note: Individual results are shown for the 34 experimental plants in Table S1.

**Table 3:** Mass in g (mean + SD) of seeds produced by different hand-pollination treatments.

Treatment	Mean Seed Mass (g)	Standard Deviation (g)	N Seeds
Cross	0.0031	0.0015	557
Self	0.0028	0.0007	14
Not manipulated	0.0042	0.0003	3
Emasculated	0.0051	–	1

## Data Availability

The data presented in this study are openly available in the FIU dataverse at https://doi.org/10.34703/gzx1-9v95/KFRE3A.

## Supplementary Materials

The following supporting information can be downloaded at: https://www.mdpi.com/article/10.3390/biology15181595/s1, Table S1: Details for individual plants in crossing experiment. Hand-pollination fruit production results. The number of flowers in each treatment that produced fruit (and did not) are shown as well as the total number of flowers receiving each treatment. % fruit set was determined by dividing the number of fruits by the number of flowers pollinated. More crosses were performed than the other treatments as we used pollen from multiple single parent plants for each cross. * Signifies significant difference at the 0.05 level, ** the < 0.0001 level, between self- and cross-pollination fruit set.

## Abbreviations

The following abbreviations are used in this manuscript:

FNAI	Florida Natural Areas Inventory
IRC	Institute for Regional Conservation
USFWS	United States Fish and Wildlife Service
