# Supplementary material for: Breeding System of Jacquemontia curtisii, an Endemic Morning-Glory of Pine Rocklands
Source: Biology (Basel). 2026 Sep 10;15(18):1595. doi: 10.3390/biology15181595 (PMC13603901; doi:10.3390/biology15181595)
Supplement: Supplementary file 1 [file biology-15-01595-s001.zip › biology-4495432-supplementary.pdf]

Table S1 – Details for individual plants in crossing experiment. Hand-pollination fruit production results. The **number** of flowers in each treatment that produced fruit (and did not) are shown as well as the total number of flowers receiving each treatment. **% fruit se**t was determined by dividing the number of fruits by the number of flowers pollinated. More crosses were performed than the other treatments as we used pollen from multiple single parent plants for each cross. * Signifies significant difference at the 0.05 level, ** the < 0.0001 level, between self- and cross-pollination fruit set.

**plant**	**cross**	**fruit**	**no fruit**	**#flrs**	**% fruit set**	**X^2_1**	**p**
1	Self	0	10	10	0		
	Cross	11	6	17	65	10.92	.0009*
	Total	11	16	27	-		
2	Self	0	10	10	0		
	Cross	17	5	22	77	16.48	.0000**
	Total	17	15	32	-		
3	Self	1	9	10	10		
	Cross	24	1	25	96	25.89	.0000**
	Total	25	10	35	-		
4	Self	0	10	10	0		
	Cross	20	2	22	91	24.84	.0000**
	Total	20	12	32	-		
5	Self	3	7	10	30		
	Cross	6	4	10	60	1.82	.1775
	Total	9	11	20	-		
6	Self	1	9	10	10		
	Cross	4	7	11	36	2.07	.1566
	Total	5	16	21	-		
7	Self	1	9	10	10		
	Cross	6	5	11	55	4.70	.0306*
	Total	7	14	21	-		
8	Self	0	10	10	0		
	Cross	31	2	33	94	33.66	.0000**
	Total	31	10	43	-		
9	Self	0	10	10	0		
	Cross	13	4	17	76	14.7	.0000**
	Total	13	14	27	-		
10	Self	0	10	10	0		
	Cross	21	2	23	91	25.11	.0000**
	Total	21	12	33	-		
11	Self	0	10	10	0		
	Cross	8	2	10	80	13.33	.0002*
	Total	8	12	20	-		
12	Self	0	10	10	0		
	Cross	26	2	28	93	29.40	.0000**

	Total	26	12	38	-		
13	Self	8	22	30	27		
	Cross	32	8	40	80	19.91	.0000**
	Total	40	30	70	-		
14	Self	0	10	10	0		
	Cross	15	5	20	75	15.00	.0001*
	Total	15	15	30	-		
15	Self	0	10	10	0		
	Cross	6	8	14	43	5.71	.0168*
	Total	6	18	24	-		
16	Self	0	10	10	0		
	Cross	11	3	14	79	14.51	.0001*
	Total	11	13	24	-		
17	Self	0	10	10	0		
	Cross	23	4	27	85	22.51	.0000**
	Total	23	14	37	-		
18	Self	1	9	10	10		
	Cross	10	6	16	63	6.95	.0084*
	Total	11	15	26	-		
19	Self	0	10	10	0		
	Cross	17	3	20	85	19.62	.0000**
	Total	17	13	30	-		
20	Self	7	11	18	39		
	Cross	36	1	37	97	24.22	.0000**
	Total	43	12	55	-		
21	Self	0	10	10	0		
	Cross	25	3	28	89	26.10	.0000**
	Total	25	13	38	-		
22	Self	0	10	10	0		
	Cross	18	3	21	86	10.35	.0013*
	Total	18	13	31	-		
23	Self	0	10	10	0		
	Cross	24	3	27	89	25.3	.0000**
	Total	24	13	37	-		
24	Self	0	10	10	0		
	Cross	4	6	10	40	5.00	.0253*
	Total	4	16	20	-		
25	Self	0	10	10	0		
	Cross	20	4	24	83	20.24	.0000**
	Total	20	14	34	-		
26	Self	0	10	10	0		
	Cross	27	1	28	96	33.31	.0000**
	Total	27	11	38	-		
27	Self	0	10	10	0		
	Cross	24	2	26	92	27.69	.0000**
	Total	24	12	36	-		

28	Self	0	10	10	0		
	Cross	19	3	22	86	21.26	.0000**
	Total	19	13	32	-		
29	Self	0	10	10	0		
	Cross	22	3	25	88	23.69	.0000**
	Total	22	13	35	-		
30	Self	0	10	10	0		
	Cross	27	2	29	93	30.26	.0000**
	Total	27	12	39	-		
31	Self	0	10	10	0		
	Cross	11	7	18	61	10.06	.0015*
	Total	11	17	28	-		
32	Self	0	10	10	0		
	Cross	5	5	10	50	6.67	.0098*
	Total	5	15	20	-		
33	Self	0	10	10	0		
	Cross	35	7	42	83	25.49	.0000**
	Total	35	17	52	-		
34	Self	0	10	10	0		
	Cross	20	4	24	83	20.24	.0000**
	Total	20	14	34	-		
TOTAL ALL PLANTS							
	Self	22	346	368	6		
	Cross	618	133	751	82	587.47	.0000**
	Total	640	479	1119	-		
